# MD Study of Solution Concentrations on Ion Distribution in a Nanopore-Based Device Inspired from Red Blood Cells

**DOI:** 10.1155/2016/2787382

**Published:** 2016-06-30

**Authors:** Yanyan Ge, Jieyu Xian, Min Kang, Xiaolin Li, Meifu Jin

**Affiliations:** College of Engineering, Nanjing Agricultural University, Nanjing 210031, China

## Abstract

A molecular dynamics model of a nanopore-based device, which is similar to the nanopores in a cell membrane, was used to determine the influence of solution concentration on radial ion distribution, screening effects, and the radial potential profile in the nanopore. Results from these simulations indicate that as the solution concentration increases, the density peaks for both the counterion and coion near the charged wall increase at different speeds as screening effects appeared. Consequently, the potential near the charged wall of the nanopore changed from negative to positive during the simulation. The detailed understanding of ion distribution in nanopores is important for controlling the ion permeability and improving the cell transfection and also the design and application of nanofluidic devices.

## 1. Introduction

The ion permeability of red blood cell membrane is associated with a variety of life activities. The hydrophilic nanopores in a cell membrane can serve as a pathway for inserting biological molecule into the cell [1, 2]. The detailed understanding of ion distribution and transport in nanopores is essential to improve the understanding and controlling of ion permeability and cell transfection [3–5]. In another way, as microfabrication techniques continue to be developed, more and more micro/nanofluidic devices have been devised. Nanofluidic devices, such as organic or inorganic pores and channels, have primary dimensions comparable to the Debye length, and so they have been wildly used for the successful separation of DNA or biomolecular sensing down to the single-molecule level [6–10]. In these devices a modulation in a baseline ion current can be observed when DNA or a biomolecule is translocated through the nanopore. Analysis of the ion current modulation can be used to gather information about the specific DNA or biomolecule of interest [11–14]. As could be expected, a detailed understanding of the device ion distribution is essential to the analysis of the ionic current signals collected during nanopore-based biosensing [12, 13, 15–18]. However, an in-depth understanding of the fundamental physics of ion and biomolecule behavior in the highly confined nanoenvironment of a nanosensor is far from complete. For example, a clear picture describing the complex interactions between the mobile ions in the solution, the surface charges, and the charges on the biomolecules themselves has yet to be put forth. Previously, only simple models have been proposed to explain the current modulation. The lack of accurate models to describe the transport laws of ions and biomolecules confined in nanofluidic channels not only has restricted the precision of the nanofluidic devices, but has also blocked them from more extensive application. Molecular dynamics (MD) simulations are a useful tool to study nanoscale fluid flow. By modeling and solving complex motion equations, the space, position, and velocity of each particle in the system can be defined. As a further step, the macroscopic quantities such as ion radial distribution, degree of screening, and potential profile can be analyzed quantitatively, providing a level of detail very difficult to arrive at experimentally. In this work, an MD model of a cylindrical nanopore 3 nm in radius was built and used to study the influence of solution concentration on the ion radial distribution, screening effects, and the potential profile of sodium chlorine solution confined in the pore. Simulation results indicated that as the solution concentration increased, the density peaks of both coion and counterion concentrations increased at different speeds as screening effects appeared. Due to the negative surface charges, the potential of the solution is negative near the charged nanopore wall but quickly becomes positive as the distance from the wall increases. Results from this simulation can be used to modify the current hydrodynamic model based on continuum theories and build an accurate mathematical model that can be used to describe the transport rules of ions and biomolecules confined in nanofluidic pores.

## 2. Details of the Molecular Dynamics Model

**Figure 1:**
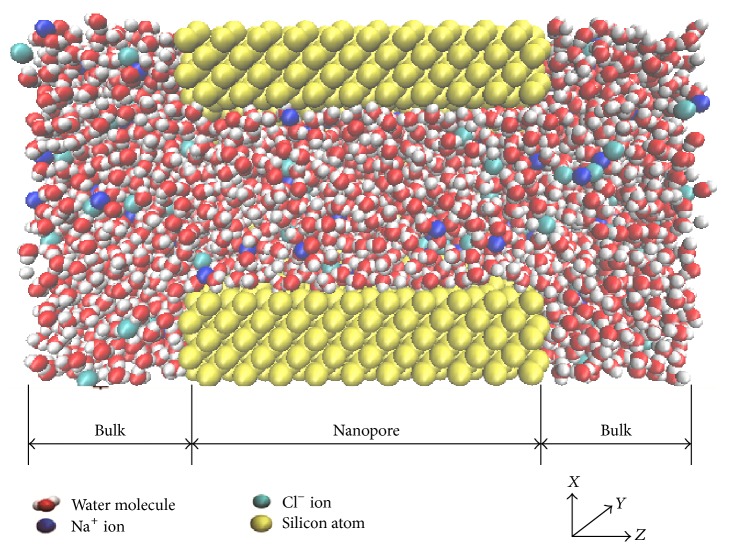
A schematic diagram of the bulk-nanopore-bulk model, which is a cross-sectional view.

A molecular dynamics model of bulk-nanopore-bulk, which is similar to a nanopore in a cell membrane, as shown in Figure 1, was modeled for different concentrations of solution using a modified TINKER 4.2 [19] MD package. The nanopore was filled with NaCl solution, with the counterions and coions randomly distributed in the solution. The initial setting of the number of coions and counterions gave the model electrical neutrality [20]. The wall of the nanopore, however, was distributed with elementary charges along the *z* direction which remained frozen to their original locations during the simulation [21]. The model and simulations included solution concentrations of 0.6 M, 1.3 M, and 2 M, with 1045 total water molecules used in the model. The Lennard-Jones (LJ) potential was used to approximate the interaction between a pair of atoms [20, 22]. The electrostatic interactions among surface charges, ions, and water molecules were modeled using the Ewald summation algorithm [22]. The water molecules themselves were modeled using SPC/E (extended simple point charge) [23].  Table 1 gives a complete list of the parameters used for the Lennard-Jones interaction in the calculation [19, 20]. The first 4 ns of the simulation were used to equilibrate the system, while the following 4 ns were used to obtain statistical data across the various solution concentrations.

Qualitatively, the wall of the nanopore is similar to a hydrophilic surface. The Steele potential is used to describe the interaction between the solid surface and the fluid molecules. The values recorded for interactions between the solid surface and the molecules within the nanopore agree well with previous results reported in the literature [20]. The Steele potential of the simulation is as follows:

(1) uwfr=2πρwεwfσwf2Δ25σwfRn−r10−σwfRn−r4−σwf43ΔRn−r+0.61Δ3,

where Δ = 2.709 Å, *ρ*_*w*_ =42.76 nm^−3^, and *ε*_*wf*_ and *σ*_*wf*_ are obtained from bulk silica parameters: *σ*_*w*_ = 3.0 Å and *ε*_*w*_/*k*_*B*_ = 230 K. The fluid molecular parameters were seen to follow the Lorentz-Berthelot rules. *R*_*n*_ is the radius of the nanopore, while *r* is the distance of the fluid molecules from the center of the nanopore. The velocity Verlet algorithm [24] was used to integrate Newton's equations of motion over 2 fs time steps. A Berendsen thermostat [25] was used to maintain the system temperature at 298.0 K with a time constant of 0.1 ps. Other simulation details can be found in a previously published paper [19].

## 3. Results of MD Simulation and Discussion

**Figure 2:**
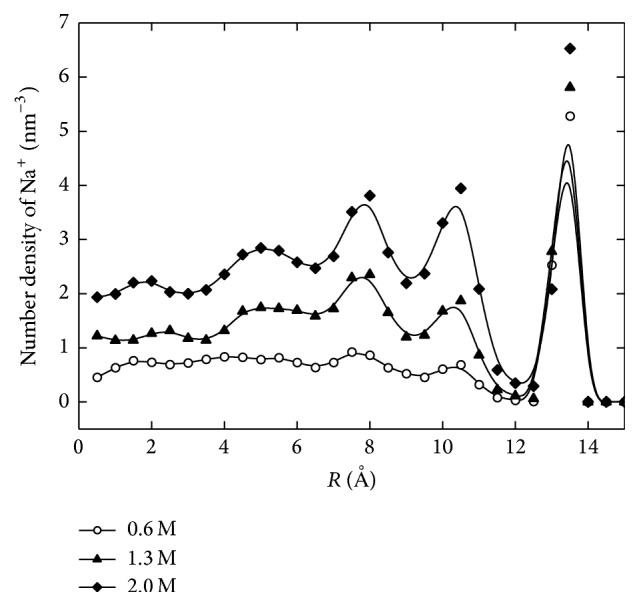
The radial distribution of various concentrations of Na^+^ ions confined in a nanopore.

**Figure 3:**
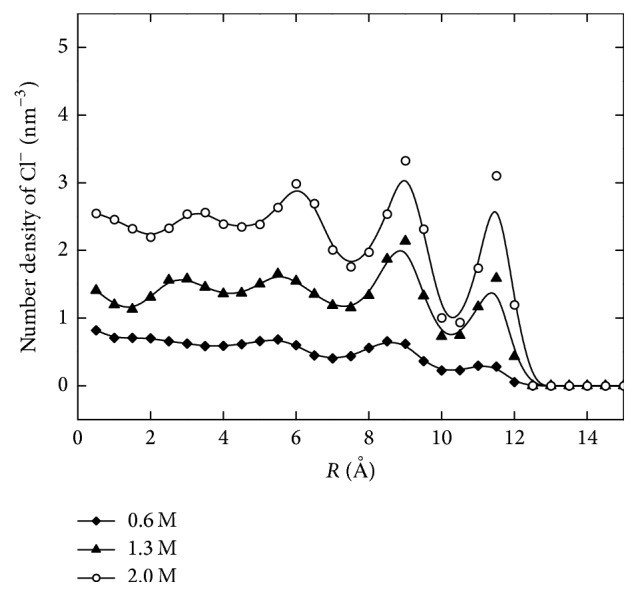
The radial distribution of various concentrations of Cl^−^ ions confined in a nanopore.

Figure 2 shows the concentration profiles of counterions along the radial direction for different solution concentrations. Due to the negative surface charges, Na^+^ ions are attracted to the wall surface while also being repelled by the Steele potential at the wall. The highest density peaks accumulated 1.5 Å away from the charged wall. As the solution concentration increased, the density peak increased in intensity while staying at the same position. This is because the negative surface charges and charge density, as well as the electrostatic interactions among the counterions, do not change.  Figure 3 shows the distribution profile of Cl^−^ ions. These negatively charged ions are repelled by surface charges on the nanopore wall, while also being attracted by the sodium ions which have relocated near the wall. Based on these forces, the peak density is located 3.5 Å away from the charged wall in the first density valley of Na^+^ ions. As the density of Na^+^ ions increases, more Cl^−^ ions are attracted and accumulated, leading to increase of both density peaks, though at different speeds. Na^+^ ion density was seen to increase more slowly than that of Cl^−^ ions. This behavior is due to the fact that near the position of the highest density the highly packed Na^+^ ions strongly repulse each other and therefore limit continued accumulation. In the case of Cl^−^ ions, the peak density increases relatively faster because it is influenced not only by the solution concentration but also by the density peak of the Na^+^ ions.

**Figure 4:**
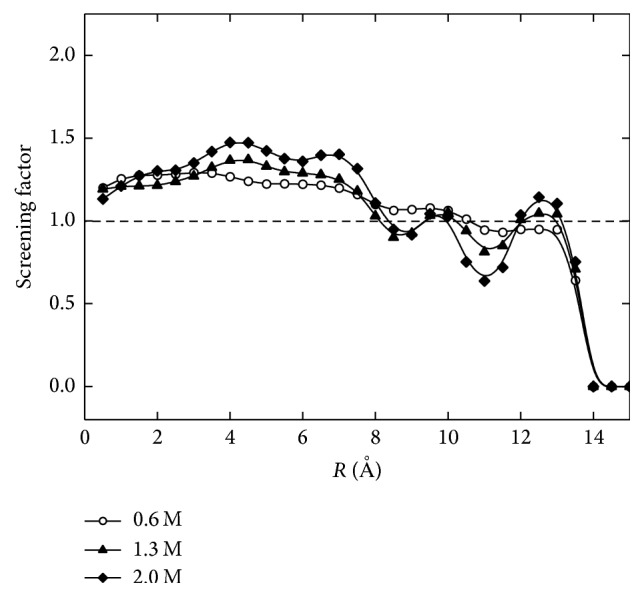
Screening factor along the radius for different solution concentrations.

**Figure 5:**
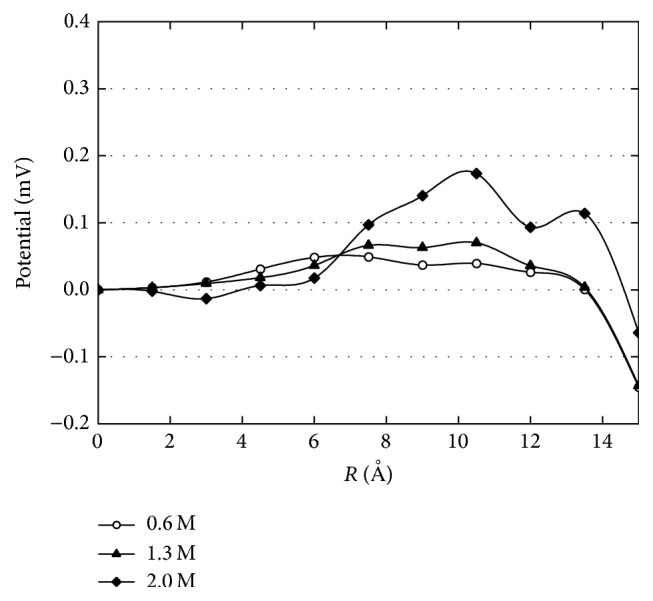
Potential profile along the radius for different solution concentrations.

Screening of the surface charge due to the ions in solution was computed using solution concentrations of 0.6 M, 1.3 M, and 2 M. The screening factor is defined as follows [14]:

(2) $$\begin{array}{c} S_{f}\left(\;r\;\right)=\frac{\int_{R}^{0}F\left[C_{Na^{+}}\left(r\right)-C_{Cl^{-}}\left(r\right)\right]dr}{\left|\sigma_{s}\right|}, \end{array}$$

where *F* is Faraday's constant, *C*_*i*_(*r*) denotes the concentration of ion *i* at the position *r*, and *σ*_*s*_ is the surface charge density. *S*_*f*_(*r*) > 1 corresponds to overscreening of the surface charge.  Figure 4 shows the screening factor given by different solution concentrations along the nanopore radius. As the solution concentration increases, the distance between the charged wall and the point where the surface charge is overscreened decreases, and the effect of thermal motion in the electric double layer becomes comparatively weaker as more ions are shielded from the surface charges. It is obvious that the shielding effect is influenced by solution concentration. The potential within the nanopore is related to both the surface charge density and the solution concentration.  Figure 5 shows the influence of solution concentration on potential. The potential is computed using Coulomb's Law and the principle of point charge superposition. The equation can be written as

(3) $$\begin{array}{c} \phi =C\overset{k}{\underset{i=1}{\sum }}\left[\;q_{i}\left(\;\frac{1}{r}-\frac{1}{r_{0}}\;\right)\;\right], \end{array}$$

in which *C* is the conversion constant, *k* is the total number of ions in solution and the elementary charge on the surface, *q*_*i*_ is the electric quantity held by ion *i* or its elementary charge, *r*_0_ is the reference point (taken as the pore center point here) at which the voltage is taken to zero, and *r* is the position of ion *i*. Because of negative surface charges the calculated potential is negative near the charged wall but quickly becomes positive as the distance away from the wall increases and Na^+^ ions have accumulated. As higher solution concentrations were used, this phenomenon becomes more evident.

## 4. Conclusions

MD simulations were used to investigate the radial ion distributions, screening factors, and potential distribution of solutions with various concentrations confined in a cylindrical nanopore. Simulation results indicate that as the solution concentration increases the density peaks of both the counterion and coion also increase, though not linearly. Because the density of surface charges remains constant, the Na^+^ ion density peak is only influenced by the solution concentration. Meanwhile the Cl^−^ ion density peak is influenced not only by the solution concentration but also by the density of Na^+^ ions. As a result, the density peak of Na^+^ ions increases more slowly than that of Cl^−^ ions. As the solution concentration increases, more sodium ions accumulate near the charged wall, making screening effects more evident. The calculated potential is related to both the charge density of the nanopore and net space charges in the diffuse part of the double layer. Due to negative surface charges, the potential is negative near the charged wall and becomes more positive away from the charged wall. These results can serve as an important reference value for the theoretical study of ion permeability and cell transfection as well as the design of nanofluidic devices used for the separation of DNA and biomolecular sensing.

## Figures and Tables

**Table 1:** Parameters for the Lennard-Jones interaction.

Pair	*σ* (Å)	*ε* (kJ·mol^−1^)
O-O	3.169	0.6502
Na^+^-O	2.876	0.5216
Cl^−^-O	3.785	0.5216
Na^+^-Na^+^	2.583	0.4184
Cl^−^-Cl^−^	4.401	0.4184
Na^+^-Cl^−^	3.492	0.4184
